# Factors Associated With Latent Classes of Exercise Motivation Among Physically Active Older Adults

**DOI:** 10.1093/geroni/igab046.2884

**Published:** 2021-12-17

**Authors:** Choi Bomi, Susanna Joo, Changmin Lee, Kwang Joon Kim, DaeEun Kim, YoonMyung Kim, Hey Jung Jun

**Affiliations:** 1 Yonsei University, Seoul, Seoul-t'ukpyolsi, Republic of Korea; 2 Yonsei University, Seodaemun-gu, Seoul-t'ukpyolsi, Republic of Korea; 3 Severance Hospital, Yonsei University College of Medicine, Seoul, Seoul-t'ukpyolsi, Republic of Korea

## Abstract

The objective of this study was to estimate the latent classes of exercise motivation and to find relevant factors in older adults. The sample comprises 179 people who reported practicing physical exercise regularly. We performed Latent Class Analysis (LCA) and multinomial logistic regression. Exercise motivation was observed with six indicators: medical advice, fun, weight loss, leisure, fitness, and socializing. Independent variables of regression analysis included sociodemographic characteristics (age, gender, marital status, education, and household income), health and well-being (subjective health, and life satisfaction), and satisfaction on neighborhood environment (physical, service, and sociocultural aspects respectively). Results of LCA indicated that the three-class model yielded optimal fit indices. Class 1 (7.5%) was labeled as ‘mainly for medical advice and socializing’. Class 2 (46.5%) was labeled as ‘mainly for fun’, while class 3 (46.0%) was labeled as ‘only fitness’. Results of multinomial logistic regression showed that males, people with lower education, and higher satisfaction with their sociocultural neighborhood were more likely to be categorized as ‘mainly for fun’ group compared to the reference group (‘only for fitness’). Subjective health was marginally significant (p<.10): People with positive subjective health tend to be categorized as ‘mainly for fun’ than ‘only for fitness’ group. Satisfaction with their sociocultural neighborhood was marginally significant (p<.10) in distinguishing ‘only for fitness’ and ‘mainly for medical advice and socializing’ group. The results of this study emphasized the heterogeneity in exercise motivation. Significant factors of exercise motivation in this study implied the importance of individualized interventions to promote exercise participation.

